# A case of revertant mosaic‐like normal‐looking spots in a patient with erythroderma with *IL36RN* and *CARD14* heterozygous mutations

**DOI:** 10.1111/1346-8138.17498

**Published:** 2024-10-07

**Authors:** Maho Matsuo, Xiaoyu Zang, Toshinari Miyauchi, Yoko Mizutani, Hirofumi Niwa, Kayoko Tanaka, Hiroaki Iwata

**Affiliations:** ^1^ Department of Dermatology Gifu University Graduate School of Medicine Gifu Japan; ^2^ Department of Dermatology, Faculty of Medicine and Graduate School of Medicine Hokkaido University Sapporo Hokkaido Japan

**Keywords:** autoinflammatory keratinization diseases, *CARD14*, erythroderma, *IL36RN*, revertant mosaicism

## Abstract

An 89‐year‐old Japanese woman presented with erythroderma associated with significant scaling. A histological examination showed acanthosis with hyperkeratosis and hyperkeratinization of the hair follicles. Genetic analyses using DNA from the peripheral blood revealed heterozygous mutations in *IL36RN* (c.115+6T>C) and *CARD14* c.2648G>A (p.Arg883His). Based on these findings, we diagnosed her with erythroderma attributable to autoinflammatory keratinization disease. She then developed more than 30 small, round, well‐defined, spots on her back and extremities that appeared histologically normal. We suspected that these spots might be revertant mosaicism. Immunohistochemical staining with p65, which is a component of nuclear factor kappa‐light‐chain‐enhancer of activated B cells (NF‐kB), revealed nuclear staining in epidermal keratinocytes in erythematous lesions, but not in the normal‐looking spots. However, mutations in *IL36RN* and *CARD14* unexpectedly persisted in the epidermis and dermis of the normal‐looking spots.

## 1 INTRODUCTION

Autoinflammatory keratinization diseases (AiKDs) represent inflammatory keratosis conditions arising from autoinflammatory mechanisms within the skin. They are characterized by inflammation affecting the superficial layers of the epidermis and dermis, along with associated abnormal keratosis.^1^, ^2^ The spectrum of AiKDs encompasses conditions such as generalized pustular psoriasis (GPP) and related disorders, pityriasis rubra pilaris (PRP) type V, and familial chronic lichenoid keratosis. Several gene mutations, including loss‐of‐function mutations in *IL36RN* and gain‐of‐function mutations in *CARD14*, have been implicated as causal factors in GPP and related diseases; gain‐of‐function mutations in *CARD14* have been specifically linked to PRP type V.^3^, ^4^ Dysfunction of the IL‐36 receptor antagonist (IL‐36Ra), encoded by *IL36RN*, initiates a downstream inflammatory cascade, while activation of CARD14, encoded by *CARD14*, promotes skin inflammation by activating NF‐κB in epidermal keratinocytes.^1^ Mutations in *CARD14* manifest clinically in various forms, including as psoriasis vulgaris (PsV), PRP, and GPP, with diverse phenotypic expressions, even from identical mutations.^5^ We present a patient with erythroderma with heterozygous mutations in *IL36RN* and *CARD14*, characterized by multiple lesions displaying what appeared to be normal‐looking spots.

## 2 CASE REPORT

**FIGURE 1:**
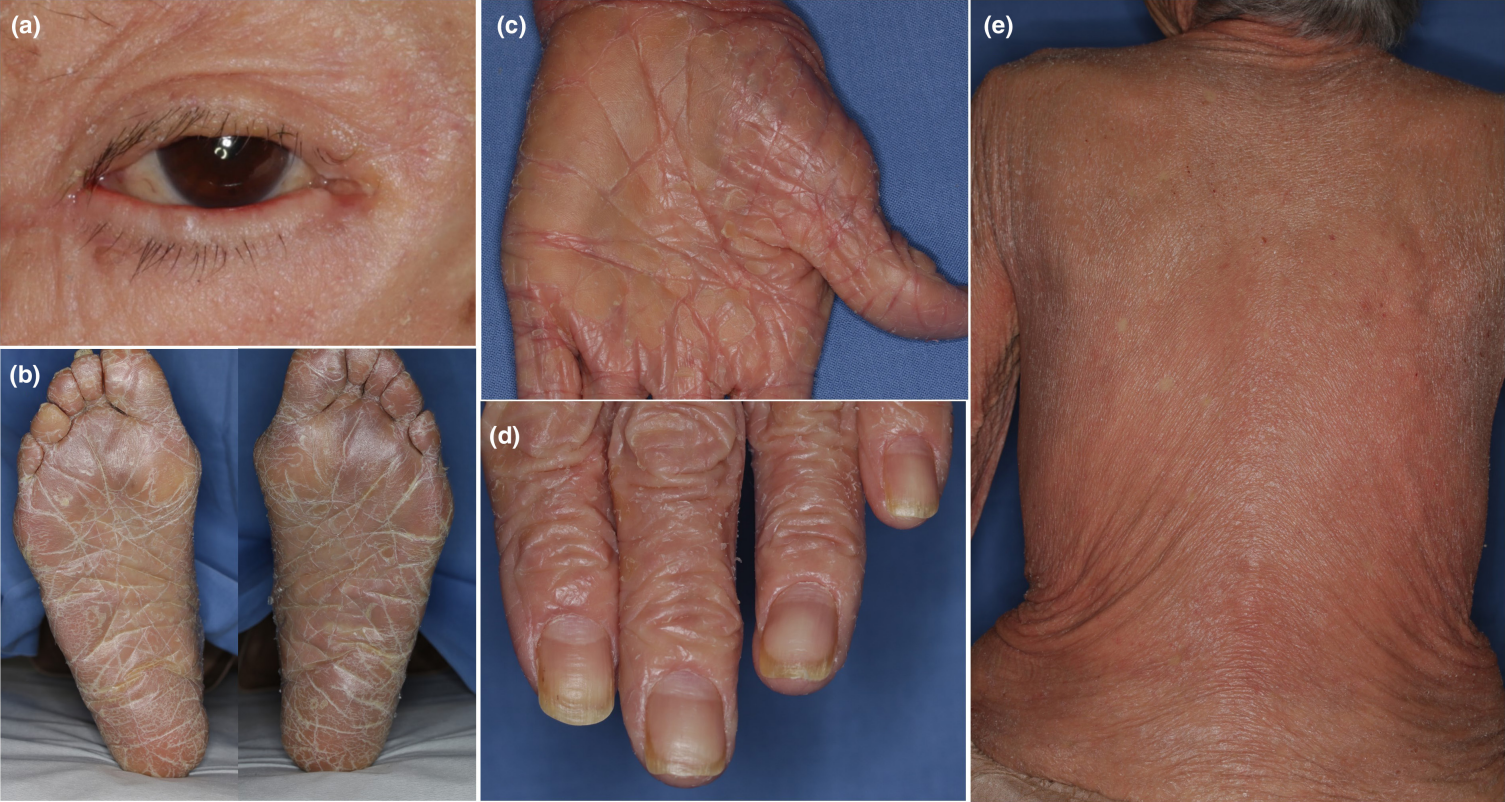
Clinical features on the skin. (a) Ectropion of her eyelids. (b) Hyperkeratosis on her soles. (c) Hyperkeratosis on her palm. (d) No changes in her nails. (e) Erythroderma on her back.

**FIGURE 2:**
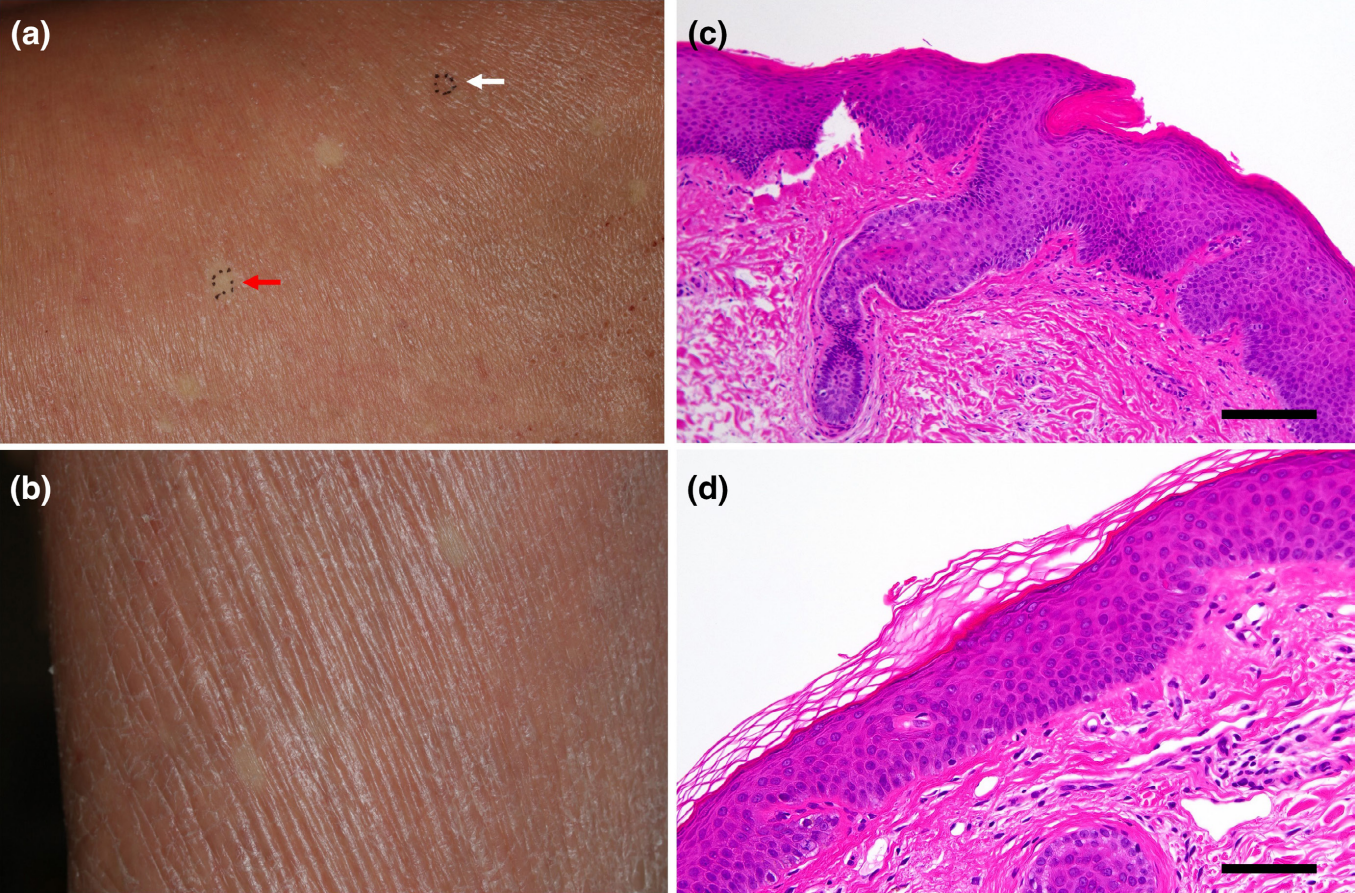
Clinical revertant spots and histology. (a) Maximum 2 cm diameter round, well‐defined‐looking normal skin spots on her back. White arrow shows area of histopathological findings in (c), and red arrow shows area of histopathological findings in (d). (b) Normal‐looking spots on the extremities. (c) Histopathological findings for thickened epidermis with hyperkeratosis and hyperkeratosis, with irregular extension of some epidermal processes, and keratinization of hair follicles in the erythematous areas (hematoxylin and eosin [H&E] staining, scale bar: 100 μm). (d) Histological findings for normal skin in normal looking skin areas (H&E staining, scale bar: 100 μm).

An 89‐year‐old Japanese woman presented with a skin rash on her upper extremities that she reported had been present since about age 5. Subsequently, erythematous rashes on the posterior aspect of the neck and the upper extremities persisted and intensified. She had no family history of dermatological conditions. PsV had been diagnosed by her local physician a decade prior to her referral to our department. She had undergone treatment with oral cyclosporine and topical steroids, and after 7 years she had observed keratinization on her palms. Following the cessation of cyclosporine due to receiving a COVID‐19 vaccination, her rash and pruritus worsened, prompting the initiation of phototherapy, which alleviated the itching but not the erythema. This led to her referral to our department. A physical examination revealed significant scaling on her scalp and face, scattered erythematous plaques on her neck and limbs, keratosis on her palms and soles, and ectropion of the eyelids (Figure 1a–c). No abnormalities were noted in her ears, nails or tongue (Figure 1d). The skin biopsy showed atypical psoriatic features, with a Psoriasis Area and Severity Index score of 20.9. Treatment with tildrakizumab was ineffective and was discontinued after two administrations. Etretinate was commenced but was stopped due to the exacerbation of dermatitis and elevated hepatic enzymes. This was followed by apremilast administration, which was also ineffective. A second skin biopsy showed epidermal thickening with hyperkeratosis and irregular epidermal processes in erythematous areas, and hyperkeratinization of the hair follicles (Figure 2c). This led to the suspicion of PRP. Genetic analyses using DNA from the patient's peripheral blood conducted by the Kazusa DNA Research Institute (Kisarazu, Japan) revealed heterozygous mutations in *IL36RN* (c.115+6T>C) (dbSNP: rs148755083) and *CARD14* c.2648G>A (p.Arg883His) (dbSNP: rs2289541). No genetic mutations were detected in *KRT1*, *KRT10*, or *KRT2*. During the disease course, pustules were never present. The resumption of etretinate led to slight improvements in her rash, although she developed erythroderma 4 months later (Figure 1e), accompanied by pruritus on her back and lower limbs. The re‐administration of tildrakizumab was, again, ineffectual, so it was discontinued after two administrations. Subsequent topical therapy with steroids and vitamin D3 provided no significant relief, and moisturizer‐only treatment did not exacerbate her condition. Throughout the course of her erythrodermic condition, more than 30 small, round, well‐defined, normal‐looking spots of up to 2 cm in diameter appeared on her back and extremities (Figure 2a,b). A histological examination of skin samples from these spots revealed a normal appearance with neither acanthosis nor hyperkeratosis (Figure 2d).

**FIGURE 3:**
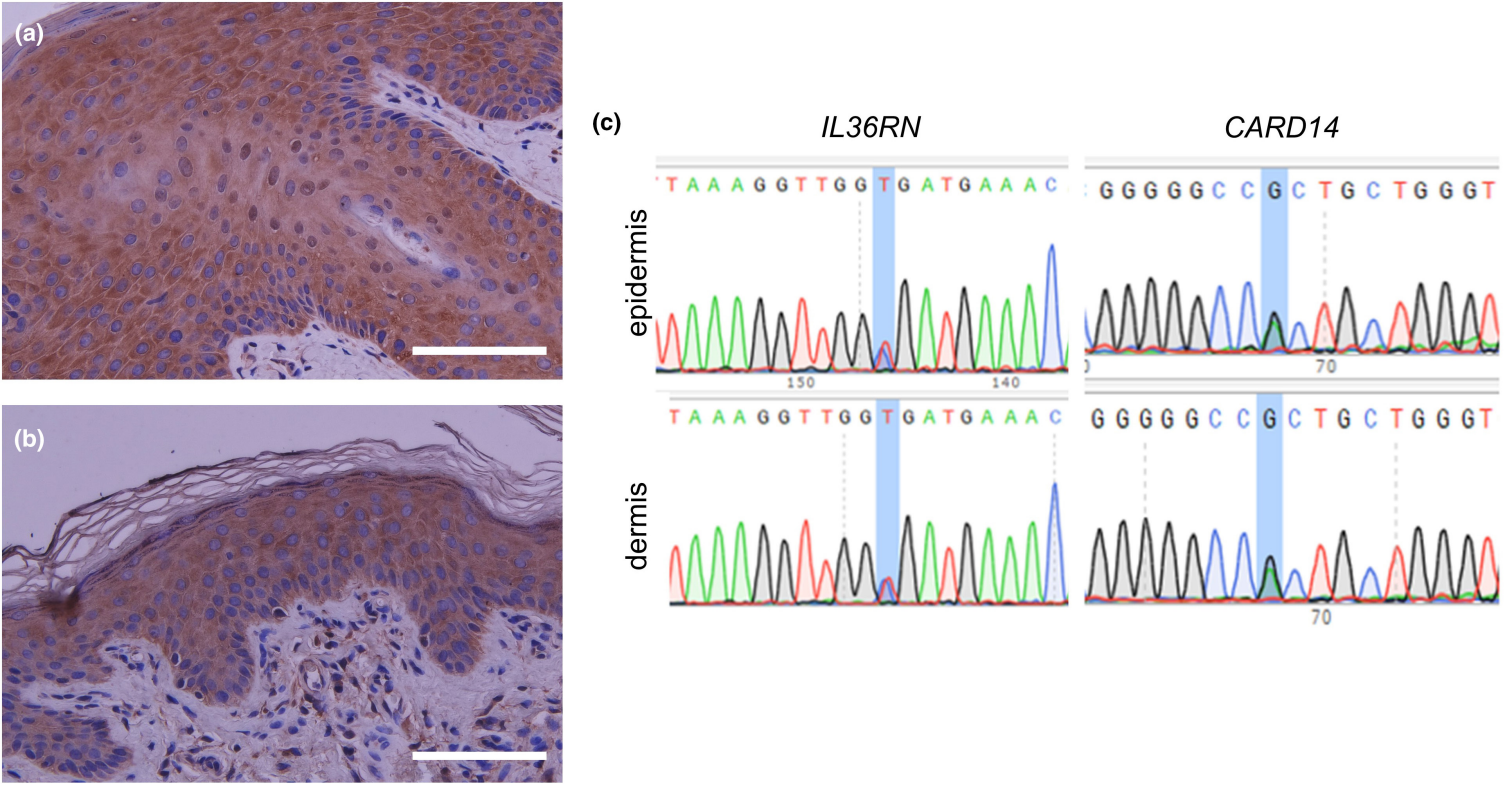
p65 staining and genetic analyses. Immunohistochemical staining of p65 demonstrated nuclear staining of epidermal keratinocytes (a) in the erythematous lesion (scale bar: 100 μm), and (b) lacking nuclear staining of epidermal keratinocytes in normal skin lesions (immunohistochemical staining of p65, scale bar: 100 μm). (c) Mutations of *IL36RN* and *CARD14* persisted in both the epidermis and dermis of the examined spots.

We suspected that these normal‐looking spots might be areas of revertant mosaicism. To confirm spontaneous gene corrections of a pathogenic mutation, we performed several tests. First, NF‐kB activity in the patient's skin was investigated by immunohistochemical staining. Staining with p65 (sc‐7151, SantaCruz,), which is a component of NF‐kB, revealed nuclear staining in epidermal keratinocytes in the erythematous lesions, but not in the normal‐looking spots (Figure 3a,b). Next, we took skin samples of 4 mm in diameter from erythematous lesions and normal‐looking areas, and we separated the epidermis from the dermis using an ammonium thiocyanate solution, as described previously.^6^ Sanger sequencing was performed using genomic DNA extracted from skin samples by means of the QIAamp DNA Mini Kit (QIAGEN). The polymerase chain reaction (PCR) primer sets were as follows: IL36RN_F GTTACTTCTGGCACAGTAGG, IL36RN_R CACTTTGCTGAGAGGTGTAG, CARD14_F TGCAGTGAGCAAAGCAGACC, and CARD14_R ATCCCCCTCCTCTGCATTCC. Unexpectedly, heterozygous mutations in *IL36RN* and *CARD14* persisted in both the epidermis and dermis of the examined spots (Figure 3c). This mutation analysis was approved by the Ethics Committee of Gifu University (no. 2023‐088).

## 3 DISCUSSION

In this case, PRP within an AiKD was suspected as the primary underlying cause of erythroderma, based on the early onset of the disease, atypical clinical and histopathological features inconsistent with PsV, and the lack of response to psoriasis therapies such as etretinate and biologic agents. The nuclear staining of NF‐kB in lesional skin and the heterozygous genetic mutations in *IL36RN* and *CARD14* were also consistent with AiKDs. Interestingly, the patient developed many clinically normal‐looking spots that were suspected of being revertant mosaicism. However, mutations in *IL36RN* and *CARD14* persisted in these normal‐looking spots.

The patient had mutations *IL36RN* and *CARD14*, but it was unclear whether either mutation was associated with her erythroderma. Heterozygous mutations in *IL36RN* have been reported in a few cases of GPP, but homozygous or compound heterozygous mutations in *IL36RN* are common. A previous report suggested that the heterozygous mutation c.115+6T>C in *IL36RN*, which induces a skip of exon 3, is associated with a mutation causative of GPP in Japanese patients.^6^ However, no reports have shown an association between this *IL36RN* mutation and PRP‐like erythroderma. Mutations in *CARD14* are known to induce a range of clinical phenotypes, including PsV, GPP, PRP, and their overlapping forms. Therefore, a CARD14‐associated papulosquamous eruption has been proposed to encompass these conditions.^7^ The heterozygous mutation c.2648G>A (p. Arg883His) in *CARD14* has been noted in PRP type I, but its pathogenicity is unknown.^8^ In silico prediction suggests this *CARD14* mutation to be benign and to have a relatively high frequency in the normal population.

Revertant mosaicism involves cells containing both mutated and mutation‐free epidermal keratinocytes that likely arise from homologous recombination in mutant cells, followed by the monoclonal expansion of mutation‐free cells.^9^, ^10^ The phenomenon of revertant mosaicism has been documented in several inherited skin diseases, including ichthyosis^10^ and epidermolysis bullosa.^11^ No instances of revertant mosaicism attributable to *IL36RN* have been documented. Since IL‐36Ra is extracellularly released as a cytokine, we anticipate that even in the event of revertant mosaicism, such mosaicism would not manifest as localized spots. In contrast, revertant mosaicism has been reported in PRP type V associated with a *CARD14* mutation.^12^ Miyauchi et al. hypothesized that *CARD14* mutations induce an aberrant response to DNA replication stress.^12^ The ensuing inflammation triggered by NF‐κB activation facilitates break‐induced replication, potentially resulting in the repair of the genetic mutation. In light of this evidence, we expected that *CARD14* could be potentially pathogenic in the present case; however, the mutation persisted even in normal‐looking spots.

We were unable to confirm the pathogenicity and genetic revertant mosaicism of mutations in *IL36RN* and *CARD14* in the present case. Several possible explanations remain for her erythroderma and clinically normal‐looking spots. In cases where both revertant and mutant keratinocytes exist in the spot, the phenotype may be normalized, and the mutation analysis should yield a mutant genotype. Other mutations in *CARD14* or in other genes may also have contributed to her condition. Sanger sequencing has difficulty detecting wide deletions, but, at least, we expect that the wide deletion of *CARD14* might not be involved in this case due to a gain‐of‐function mutation in *CARD14* for AiKDs. Therefore, some other gene mutations may have contributed to her condition.

In conclusion, the clinical findings lead us to strongly suspect revertant mosaicism in this patient with erythroderma, although definitive proof remains elusive. We are still investigating the reason for the clinically normal‐looking spots in this case and the mechanism behind them, and we will pursue further investigations in the future.

## CONFLICT OF INTEREST STATEMENT

None declared.
